# Medical History from the Earliest Times

**Published:** 1892-08-13

**Authors:** E. T. Withington


### Medical History from the Earliest Times.

By E. T. Withington, M.A., M.B. Oxon.

XXI.—THE MEDICAL PROFESSION IN ROME.

In 1723 the annual Harveian oration, delivered by Dr. Richard Mead, dealt with the early history of medicine, and gave rise to a controversy on the social state of physicians in Rome which spread throughout Europe, and lasted so long that industrious Germans have devoted special works to its history. But it will be sufficient for us to accept the judgment of Cicero, who says that medicine, like architecture, is no dishonourable occupation "for those to whose rank in life it is suited." Had the great orator been asked whom he meant, he would probably have answered, with some surprise, "Freedmen and foreigners; certainly no Roman citizen." Recollecting his own physician, Asclepiades, he might have added that other nations had other ideas, and that should a cultured and travelled Greek come to Rome, he (the consul) would not hesitate to meet him as an equal, though a physician, and might even invite him to the Tusculan villa to discuss Falernian wine and the Epicurean philosophy with Licenius Crassus and other consulars. But that his own son, or the son of any other respectable Roman, should study medicine, except purely as an amateur—as that intelligent young man, Cornelius Celsus, seemed to be doing—would be a calamity *quam avertant Dei immortales*. Crassus, indeed, was dead when Cicero became consul, and Celsus was probably yet unborn, but the above may be taken as expressing the ideas of even the more liberal-minded Republicans on the status of the physician.

In later times, when Julius had granted the citizenship; when Augustus had twice owed his life to his physicians; when Tiberius and Nero had well nigh destroyed the old aristocracy of Rome, and slaves and freedmen had the ear of the emperors, the social position of medicine was greatly altered, and we find Quintilian recommending as a subject for school essays a question which would probably have much disgusted the consul, whose eloquence saved his country, "Which is the most useful member of the State, the orator, the physician, or the philosopher?" Two other causes contributed to this rise of medicine in the social scale, the establishment of the archiatrate, which deserves a chapter to itself, and the wealth acquired by some of its practitioners. We must not exaggerate the latter; the case was then much as it is now. A few able practitioners, a few notorious quacks, might obtain great possessions. Xenophon might receive £5,000 yearly from an emperor and complain that he could have got more in private practice; Charmis, the water doctor from Marseilles, might be paid £2,000 by a single patient; but there was the usual crowd at the bottom of the ladder, and we hear of several physicians who were compelled to take to less honourable but more lucrative callings to escape starvation, one of whom, to the delight of the wits of the age, became an undertaker's assistant. A large proportion of the profession, too, still belonged to the servile class. Nor is the reason far to seek, for, by having one or more slaves educated as physicians, their owner not only saved medical expenses, but greatly increased the value of his property, and the Justinian code estimates the *servus medicus* at 60 solidi (£30), just three times the value of an ordinary labourer, and double that of an artisan.

The development of specialism, and of those various medical parasites who naturally flourish in large and luxurious communities is perhaps the most striking characteristic of professional history under the early emperors. The skirts of the medical robe have always had an unfortunate tendency to drag in the dirt, and the general character of many of these specialists was anything but reputable. Only the more prominent classes can be here mentioned, and we shall first consider the ophthalmic surgeons and drug sellers, leaving the lady doctors and medical rubbers for a future article.

In the "De Oratore," Cicero makes Crassus observe that all arts are degraded by division; and he asks ironically, "Do you suppose that in the days of the Coan Hippocrates there were special physicians for diseases, others for wounds, and others, again, for the eyes?" We have seen that there certainly were, at any rate in Egypt, and in Greece we hear both of surgeons and ophthalmists very soon after the Hippocratic age, though it was not till the Roman period that specialism became prominent. Galen tells us that, in his time, large cities, such as Rome and Alexandria, swarmed with specialists, who also travelled about from place to place. Martial enumerates some of them in an epigram, "Cascellius extracts and repairs bad teeth; you, Hyginus, cauterise ingrowing eyelashes; Faunius cures a relaxed uvula without cutting; Eros removes brand-marks from slaves; Hermes is a very Podalirius for ruptures." But the most numerous and notorious were the oculists, who seem to have been further divided into a medical-surgical branch. The great occupation of the former was the invention of new forms of eye-salve, which they stamped with their names, and nearly 200 seals used for this purpose have been found scattered throughout the empire. The frequent defective spelling of these seals is only one of the many proofs of the bad education of the class. Thus Galen, writing on diseases of the eye, says he thought it useless to treat the subject scientifically, for the oculists would not understand it. In cases of corneal abscess it was their custom to seize the patient's head and shake it till the abscess burst, a treatment which, according to Galen, was often successful.

The satirists are peculiarly bitter in their attacks on this class of specialists. "Now you are a gladiator who once were an ophthalmist," writes Martial; "you did as a doctor what you do as a gladiator." Another epigram is yet severer. "The blear-eyed Hylas would have paid you sixpence, O, Quintus; one eye is gone, he will still pay threepence; make haste and take it, brief is your chance, when he is blind he will pay you nothing." But we must not suppose that there were no good eye doctors. Demosthenes, of Marseilles, whose great work on ophthalmic surgery still existed in the Middle Ages, is always mentioned with respect; others held official positions in the Roman fleets and armies, and we are surprised by the curiously-sounding title, "Oculist to the British Navy." It has even been suggested that one who is, in a sense, the greatest of physicians, St. Luke the Evangelist, was a specialist of this class, and that he travelled with St. Paul for the purpose of looking after that affection of the eyes with which the great Apostle is believed to have been afflicted.

The Romans used the title "medicus," as we that of "doctor," with great liberality, and sometimes applied it to a class of persons whom the Greeks called "drug-sellers" (pharmacopolæ), who were the middle-men between the physicians and the herb gatherers (rhizotormi). The ancient physician compounded his own prescriptions, though Pliny reproaches those of his day with being unable to do so, and having to buy them ready-made from the drug-seller; and we must remember that it was not till the Arabic period that the drug-seller developed into the apothecary, or middle-man, between the physician and his patient. But doubtless they often sold medicines to the public, as well as perfumes, poisons, love philtres, &c., and they certainly managed to earn a very bad reputation. We find them classed with actors, ballet girls, and other (at that time) disreputable persons. A demagogue is said to bear the same relation to a genuine politician as a drug-seller does to a doctor; and a writer seeking for a type of utter degradation says it is as though a good physician became a drug-seller. The chief charge against them is indicated by the fact that "medicamentarius," which in the days of Pliny meant "drug-seller," had, by the time of Theodosius, become the recognised word for "poisoner."

On the other side, we may mention the last will and testament of a drug-seller in the small town of Larinum, who bequeathes 300 pots of drugs, and about £600 in money, to his son-in-law, on condition that he shall always supply the poor with medicines gratis. "He must have been a Christian," say admiring commentators; but we may hope that even a Pagan might rise to that height of *post-mortem* benevolence.

